# Characterization and Phylogenetic Analysis of the Complete Mitochondrial Genome of *Laelia suffusa* (Lepidoptera: Erebidae, Lymantriinae)

**DOI:** 10.1093/jisesa/ieaa138

**Published:** 2021-01-11

**Authors:** Jing Li, Qing Lv, Xiao-man Zhang, Hui-lin Han, Ai-bing Zhang

**Affiliations:** 1 College of Life Sciences, Capital Normal University, Beijing, P. R. China; 2 School of Forestry, Northeast Forestry University, Harbin Heilongjiang, P. R. China

**Keywords:** *Laelia suffusa*, Lepidoptera, Erebidae, mitogenome, phylogeny

## Abstract

In this study, the complete mitochondrial genome of a white tussock moth, *Laelia suffusa* (Walker, 1855) (Lepidoptera: Erebidae, Lymantriinae), was sequenced and annotated. The genome sequence was 15,502 bp in length and comprised 13 PCGs, 2 *rRNAs*, 22 *tRNAs*, and a single noncoding control region (CR). The nucleotide composition of the genome was highly A + T biased, accounting for 79.04% of the whole genome and with a slightly positive AT skewness (0.015). Comparing the gene order with the basal species of Lepidoptera, a typical *trnM* rearrangement was detected in the mitogenome of *L. suffusa*. Besides, the *trnM* rearrangement was found at the head of *trnI* and *trnQ,* rather than at the back. The 13 PCGs used ATN as their start codons, except for the *cox1* which used CGA. Out of the 22 *tRNAs*, only 1 *tRNA* (*trnS1*) failed to fold in a typical cloverleaf secondary structure. The conserved motif ‘ATAGA + poly-T’ was detected at the start of the control region which was similar to other Lepidoptera species. In total, 10 overlapping regions and 19 intergenic spacers were identified, ranging from 1 to 41 and 2 to 73 bp, respectively. Phylogenetic analysis showed that Lymantriinae was a monophyletic group with a high support value and *L. suffusa* was closely related to tribe Orgyiini (Erebidae, Lymantriinae). Moreover, the phylogenetic relationship of Noctuoidea (Lepidoptera) species was reconstructed using two datasets (13 PCGs and 37 genes) and these supported the topology of (Notodontidae + (Erebidae + (Nolidae + (Euteliidae + Noctuidae)))).

Lepidopteran, including moths and butterflies, are globally distributed phytophagous insects and one of the four largest holometabolous orders (Kristensen et al. 2007). More than 180,000 species of Lepidoptera are described and are only second in the class Insecta (Scoble 1992, Pogue 2009, Mullen and Zaspel 2019). *Laelia* (Lepidoptera: Erebidae, Lymantriinae) is a genus of tussock moths in the family Erebidae, whose distribution spans throughout Europe and Asia (Chao 1987, 2003). *Laelia suffusa* is a white tussock moth that is mainly distributed in the south and southeast of Asia (Chao 1987, Ahmed et al. 2002). Besides, it is regarded as one of the main pasture and rice fields pests and can cause significant economic losses if not well managed, which are associated with its larvae behavior, short life cycle, population explosion, and can have six to seven generations per year (Chao 1987, Ahmed et al. 2002). A high population of *L. suffusa* can consume leaves from culms and tillers regardless of leaf age or plant (Ahmed et al. 2002). The larvae are covered with yellow hairs on their backs and can cause cutaneous eruptions outbreaks (Dubois and Dean 1995, Yanar et al. 2017). Previous studies have focused on species diversity (Fox 2013, Ju et al. 2016, Singh and Navkiran 2019, Zhang et al. 2019), pest control (Li and Yan 1991, Armstrong et al. 2003, Yu et al. 2019, Sun et al. 2020), species identification and genetic variations (Singh and Navkiran 2019), and insect physiological adaption (Zapletalová et al. 2016) of *Laelia*. However, very limited research has been conducted on the phylogenetic relationship of genus *Laelia*. Considering that *L. suffusa* is an important pest of rice in Asia, it is important to identify its classification using molecular approaches. This information might provide further understanding of the origin and genetic differentiation with other Lepidoptera pests and a basis for biological pest control.

Phylogenetic hypothesis of the superfamily Lepidoptera revealed that there were six strongly supported clades: Noctuidae, Erebidae, Notodontidae, Euteliidae, Nolidae, and Oenosandridae (Zahiri et al. 2011). Previously, Lymantriidae was considered as a family-level classification unit, however, based on morphological and molecular evidence, it was classified as a subfamily of Erebidae (Mitter et al. 2017, Regier et al. 2017). However, the phylogenetic relationships within Lymantriinae are still under active debate. Initially, it was believed that five tribes were included in the subfamily Lymantriinae: Arctornithini, Lymantriini, Leucomini, Nygmiini, and Orgyiini (Zahiri et al. 2011, Zahiri et al. 2012, Wang et al. 2015). However, based on the phylogenetic analysis using several mitochondrial and nuclear genes, the phylogenetic relationship within Lymantriinae could be illustrated as follows: Arctornithini was a sister group to Orgyiini + Nygmiini together with Lymantriini + Leucomini (Zahiri et al. 2011, 2012; Wang et al. 2015). However, the phylogenetic relationship based on various molecular markers and taxonomy has often been found to be conflicting, leading to a serious debate on their relative positions (Fibiger and Lafontaine 2005, Mitchell et al. 2006).

Owing to some unique features as maternal inheritance, rapid mutation rate, and limited recombination compared with nuclear genes, mitochondrial DNA markers, and mitogenome have been extensively used in phylogenetic analysis, comparative genomics studies, and species identification (Avise 2009, Cameron, 2014, Qin et al. 2019, Tyagi et al. 2020). The insect mitogenome has a relatively stable structure that encodes 37 genes and a noncoding control region scattered on the circular DNA molecule and with a length of 15,000–18,000. The 37 genes generally include 13 protein-coding genes (PCGs): cytochrome c oxidase genes (*cox1, cox2*, and *cox3*), NADH dehydrogenase genes (*nad1, nad2, nad3, nad4, nad4L, nad5*, and *nad6*), cytochrome B genes (*cytb*), ATPase genes (*atp6* and *atp8*), 22 transfer RNA genes (*tRNA*s), and 2 ribosomal RNA genes (*rRNA*s, *rrnL* and *rrnS*). Besides, a large noncoding region controls replication and transcription (CR region, also named A + T-rich region; Wolstenholme 1992, Ballard and Whitlock 2004, da Fonseca et al. 2008, Cameron 2014). Compared with the single molecular markers from plastids and the nuclear genome, mitogenome can provide information on evolutionary and speciation events required in insect phylogenetic studies (Boore 2006, Qin et al. 2019). Recently, the rapid development of sequencing technologies such as the next-generation sequencing technology has triggered an increase in the available mitochondrial genome data (Cameron 2014).

In this study, the complete mitogenome of *L. suffusa* was sequenced, annotated, and characterized. Using the newly sequenced mitogenome, phylogenetic relationships of Noctuoidea were reconstructed using both the 13 PCGs and whole genomes. The mitogenome of *L. suffusa* provided information for future research on species identification, diversity conservation, and population genetics of Erebidae and Noctuoidea.

## Materials and Methods

### Sampling and DNA Extraction

The specimens of *L. suffusa* were collected from Mt. Luofu, Guangdong Province, P. R. China, and identified based on morphological characteristics. All the collected samples were preserved in 95% ethanol in the field and stored at −20°C. Total genomic DNA was extracted from the leg muscle tissue using the Genomic DNA Extraction Kit (QIAGEN, Hilden, Germany) following the manufacturer’s instructions.

### Mitogenome Sequencing, Assembly

The purified DNA samples were used to prepared an Illumina TruSeq library and sequenced on the Illumina HiSeq2500 platform. The sequencing platform generated a total of 15-Gb paired-end reads of 150-bp length. Trimmomatic software was used to read trimming the adapters (Fragkostefanakis et al. 2012, Bolger et al. 2014). NGSQC-Toolkit v2.3.336 software and Prinseq were used to conduct rapid quality control of the raw reads. Low quality (ambiguous bases) and short reads (shorter than 95 bp) were filtered during quality control (Schmieder and Edwards 2011). Finally, high-quality reads not less than 8 Gb were adopted in the de novo assembly and annotation using MitoZ software (Meng et al. 2019). Sequences from the *cox1, cytb* and *rrnS* fragments of *L. suffusa* were used as checkout markers for mitogenome assemblies and they were amplified and sequenced using PCR and Sanger sequencing, respectively.

### Mitogenome Analysis

MEGA v8.0 was used to analyze the base composition and codon usage of the *L. suffusa* mitogenome (Kumar et al. 2016). Besides, the start and stop codons of the protein-coding genes were identified and validated by comparing with mitogenomes of other Erebidae species. tRNAscan-SE v1.21 software was used to predict the secondary structures of the 22 *tRNA* genes (Lowe and Chan 2016). The AT and GC asymmetry were represented by the values of AT-skew and GC-skew, calculated as follows: AT-skew = (A−T)/(A+T) and GC skew = (G−C)/(G+C) (Perna and Kocher 1995). The Tandem Repeats Finder program was used to predict the tandem repeats of the control region using the default parameters (Benson 1999).

### Phylogenetic Analysis

The phylogenetic tree of 52 lepidopteran species (25 species of Erebidae, 19 species of Noctuidae, 2 species of Nolidae, 3 species of Notodontidae, single species of Euteliidae, and 2 outgroups) was reconstructed to confirm the phylogenetic position of the genus *Laelia* within the superfamily Noctuoidea (Table 1). We aligned nucleotide sequences of the 13 PCGs with MAFFT. The nonprotein coding regions were aligned using MUSCLE and default parameters were used (Edgar 2004). SequenceMatrix was used to concatenate the separated genes and partitions (Vaidya et al. 2011). The concatenated sets of nucleotides were organized into two datasets: one dataset included the 13 PCGs while the other represented all the 37 genes (13 PCGs, 22 *tRNAs*, and 2 *rRNAs*). DAMBE was used to examine the substitution saturations of the two datasets (Xia and Xie 2001). Both datasets were used to perform phylogenetic analyses using maximum likelihood (ML) and Bayesian inference (BI) (Ronquist and Huelsenbeck 2003). ML and BI analyses were performed using RAxML v7.9.6 and MrBayes v 3.2.2, respectively (Stamatakis 2006, Ronquist et al. 2012). The GTR+G+I model was selected in the two datasets and 1,000 bootstrap replicates were used in RaxML. BI analysis was conducted using four independent Markov chains were set to run for 10 million generations with sampling every 1,000 generations. The first 25% of the sampled trees were discarded as burn-in. All runs were stable after 10 million generations, determined by Tracer v1.5.0. FigTree v1.4.2 was used to visualize the topologies of the phylogenetic trees (Rambaut 2014).

**Table 1.** Taxonomy, GenBank accession numbers, mitogenome sizes, and related information of 52 moths mitochondrial genomes used for the phylogenetic analysis

Superfamily	Family	Subfamily		GBAN	Length	A+T%	AT-skew	GC-skew
Noctuoidea	Erebidae	Lymantriinae	***Laelia suffusa***	**MN908152**	**15,502**	**79.0**	**0.015**	**−0.294**
			*Lymantria dispar*	FJ617240	15,569	79.9	0.016	**−**0.248
			*Lymantria umbrosa*	KY923066	15,642	79.9	**−**0.015	0.251
			*Dasorgyia alpherakii*	KJ957168	15,755	81.4	0.004	**−**0.263
			*Euproctis pseudoconspersa*	KJ716847	15,461	79.9	0.011	**−**0.242
			*Euproctis similis*	KT258910	15,437	80.2	0.000	**−**0.243
			*Gynaephora menyuanensis*	KC185412	15,740	81.5	0.003	**−**0.270
			*Gynaephora minora*	KY688086	15,801	81.5	0.006	**−**0.269
			*Gynaephora jiuzhiensis*	KY688085	15,859	81.5	0.003	**−**0.272
			*Gynaephora ruoergensis*	KY688083	15,803	81.5	0.007	**−**0.273
			*Gynaephora qumalaiensis*	KJ507134	15,753	81.4	0.007	**−**0.268
			*Gynaephora qinghaiensis*	KJ507133	15,747	81.3	0.005	**−**0.271
			*Gynaephora aureata*	NC029162	15,773	81.4	0.006	**−**0.272
		Aganainae	*Asota plana lacteata*	KJ173908	15,416	80.3	**−**0.002	**−**0.238
		Arctiinae	*Amata formosae*	KC513737	15,463	79.5	**−**0.027	**−**0.266
			*Lemyra melli*	NC026692	15,418	78.7	0.001	**−**0.225
			*Callimorpha dominula*	KP973953	15,496	81.0	**−**0.011	**−**0.201
			*Vamuna virilis*	KJ364659	15,417	80.4	0.000	**−**0.229
			*Nyctemera albofasciata*	KM244681	15,295	80.8	**−**0.015	**−**0.227
			*Cyana sp.*	KM244679	15,494	81.2	**−**0.014	**−**0.223
			*Hyphantria cunea*	GU592049	15,481	80.4	0.010	**−**0.230
		Calpinae	*Eudocima phalonia*	NC032382	15,575	80.7	**−**0.013	**−**0.219
		Erebinae	*Catocala deuteronympha*	KJ432280	15,671	81.1	**−**0.022	**−**0.231
			*Grammodes geometrica*	KY888135	15,728	80.5	**−**0.003	**−**0.220
		Hypeninae	*Paragabara curvicornuta*	KT362742	15,532	80.4	**−**0.008	**−**0.226
	Noctuidae	Noctuinae	*Sesamia inferens*	JN039362	15,413	80.2	**−**0.001	**−**0.230
			*Spodoptera frugiperda*	KM362176	15,365	81.1	0.004	**−**0.192
			*Spodoptera litura*	JQ647918	15,388	81.0	0.013	**−**0.195
			*Spodoptera exigua*	JX316220	15,365	80.9	0.010	**−**0.195
			*Agrotis ipsilon*	KF163965	15,377	81.3	**−**0.006	**−**0.177
			*Agrotis segetum*	KC894725	15,378	80.7	**−**0.004	**−**0.192
			*Noctua pronuba*	KJ508057	15,315	81.1	**−**0.018	**−**0.170
			*Striacosta albicosta*	KM488268	15,553	79.3	0.012	**−**0.238
			*Athetis lepigone*	MF152842	15,589	81.3	**−**0.024	**−**0.188
			*Mythimna separata*	KM099034	15,332	81.0	**−**0.012	**−**0.193
			*Protegira songi*	KY379907	15,410	80.2	0.000	**−**0.206
		Heliothinae	*Helicoverpa punctigera*	KF977797	15,382	81.4	0.001	**−**0.187
			*Helicoverpa zea*	KJ930516	15,343	81.0	0.002	**−**0.202
			*Helicoverpa assulta*	KR149448	15,373	80.8	**−**0.003	**−**0.191
			*Chloridea subflexa*	KT598688	15,323	80.7	0.002	**−**0.189
			*Helicoverpa armigera*	GU188273	15,347	81.0	0.001	**−**0.192
		Plusiinae	*Ctenoplusia limbirena*	KM244665	15,306	81.0	**−**0.036	**−**0.174
			*Ctenoplusia agnata*	KC414791	15,261	81.1	**−**0.024	**−**0.185
		Acronictinae	*Acronicta psi*	KJ508060	15,350	79.1	0.034	**−**0.246
	Euteliidae	Euteliinae	*Eutelia adulatricoides*	KJ185131	15,360	80.9	**−**0.006	**−**0.184
	Nolidae	Chloephorinae	*Gabala argentata*	KJ410747	15,337	81.7	**−**0.030	**−**0.174
		Risobinae	*Risoba prominens*	KJ396197	15,343	81.1	**−**0.007	**−**0.176
	Notodontidae	Pygaerinae	*Clostera anachoreta*	KX108766	15,456	80.7	**−**0.019	**−**0.217
		Phalerinae	*Phalera flavescens*	JF440342	15,659	80.9	**−**0.009	**−**0.177
		Thaumetopoeinae	*Ochrogaster lunifer*	AM946601	15,593	77.8	0.030	**−**0.318
Pyraloidea	Crambidae		*Chilo suppressalis*	NC015612	15,395	80.7	0.008	**−**0.235
Tortricoidea	Tortricidae		*Adoxophyes honmai*	NC008141	15,680	80.4	**−**0.001	**−**0.196

Note: *Nyctemera arctata albofasciat* was used to in sequence of KJ173908, which was modified to *Nyctemera albofasciat* according to subsequent revision of species list. Similarly, *Heliothis subflexa* of KT598688 was amended to *Chloridea subflexa*, and *Lachana alpherakii* was modified to *Dasorgyia alpherakii*. The species in bold was first sequenced in this study.

## Result

### Genome Organization and Base Composition

**Fig. 1.**
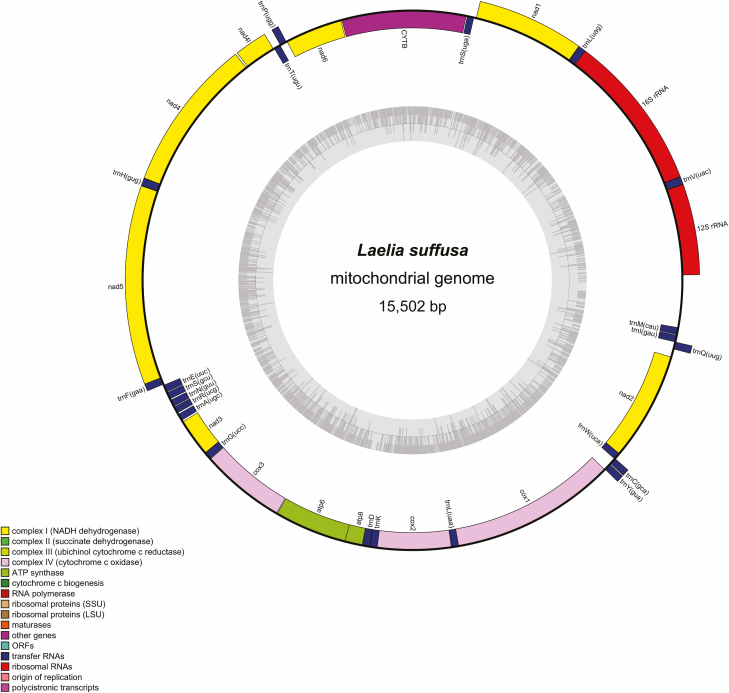
Circular map of the *Laelia suffusa* mitogenome. Protein coding and ribosomal genes are shown with standard abbreviations. *tRNA* genes are exhibited as single-letter abbreviations, except for the S1 = AGN, S2 = UCN, L1 = CUN, and L2 = UUR. The thick lines outside the circle indicate the major strand, whereas those inside the circle indicate the minor strand.

**Fig. 2.**
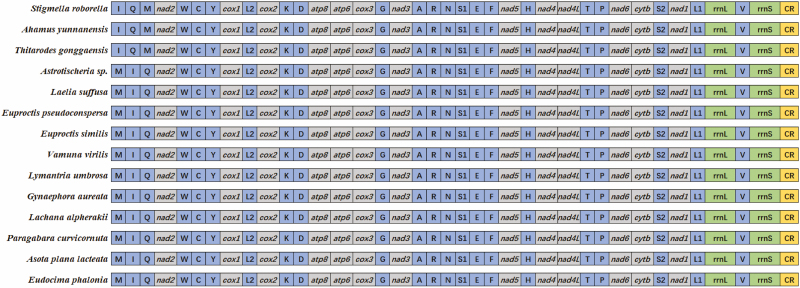
The gene order of *Laelia suffusa* mitogenome and other 13 lepidopteran species.

The *L. suffusa* mitogenome was a typical circular DNA molecule of 15,502 bp in length (GenBank MN908152; Table 1; Fig. 1). The newly sequenced mitogenome coded the 37 genes, 13 PCGs, 22 *tRNA*s, and 2 *rRNA*s (small ribosomal RNA [*rrnS*] and large ribosomal RNA [*rrnL*]), and the A+T-rich noncoding region (Table 2). In total, 23 genes (9 PCGs and 14 *tRNA*s) were transcribed on the major strand (J-strand) and the remaining 14 genes were transcribed on the minor strand (N-strand). There were 9 genes overlapping regions and 19 noncoding regions in the mitogenome of *L. suffusa*. Besides the control region, the largest noncoding region was located between gene *trnQ* and *nad2*, which was 73 bp in length (Table 2). The gene order of the genome was identical to that previously published of Lymantriidae mitogenome sequences (Fig. 2). The nucleotide composition of the *L. suffusa* sequence was highly A + T biased: A = 6,218 (40.11%), G = 1,147 (7.40%), T = 6,035 (38.93%), and C = 2,102 (13.56%) (Table 3). Furthermore, the mitogenome showed a was slightly positive AT-skew (0.015), illustrating there were more As than Ts. The GC-skew was negative (−0.294), declaring a higher frequency of base C than G when compared with other species of Noctuoidea (Table 1).

**Table 2.** Annotation and gene organization of the *Laelia suffusa* mitogenome

Gene	Strand	Nucleotide number	Size (bp)	Intergenic nucleotides	Anticodon	Start codon	Stop codon
*trnM*	J	1–67	67	4	CAU	–	–
*trnI*	J	72–137	66	**−**3	GAU	–	–
*trnQ*	N	135–203	69	73	UUG	–	–
*nad2*	J	277–1,290	1014	±2	–	ATT	TAA
*trnW*	J	1,289–1,354	66	**−**8	UCA		
*trnC*	N	1,347–1,411	65	6	GCA		
*trnY*	N	1,418–1,482	65	**−**41	GUA		
*cox1*	J	1,442–3,020	1579	3	–	CGA	T
*trnL2(UUR)*	J	3,016–3,082	67	0	UAA		
*cox2*	J	3,083–3,748	702	16	–	ATA	T
*trnK*	J	3,765–3,835	71	**−**1	CUU		
*trnD*	J	3,835–3,902	68	0	GUC		
*atp8*	J	3,903–4,067	165	**−**7	–	ATA	TAA
*atp6*	J	4,061–4,,738	678	4	–	ATG	TAA
*cox3*	J	4,743–5,531	789	2	–	ATG	TAA
*trnG*	J	5,534–5,601	68	0	UCC		
*nad3*	J	5,602–5,955	354	14	–	ATA	TAA
*trnA*	J	5,970–6,035	66	12	UGC		
*trnR*	J	6,048–6,112	65	5	UCG		
*trnN*	J	6,118–6,182	65	10	GUU		
*trnS1(AGN)*	J	6,193–6,259	67	5	GCU		
*trnE*	J	6,265–6,333	69	6	UUC		
*trnF*	N	6,340–6,406	67	**−**20	GAA		
*nad5*	N	6,387–8,147	1761	0	–	ATA	TAA
*trnH*	N	8,148–8,215	68	**−**1	GUG		
*nad4*	N	8,215–9,557	1343	14	–	ATG	TA
*nad4l*	N	9,572–9,859	288	6	–	ATG	TAA
*trnT*	J	9,866–9,929	64	0	UGU		
*trnP*	N	9,930–9,994	65	4	UGG		
*nad6*	J	9,999–10,529	531	7		ATG	TAA
*cytb*	J	10,537–11,688	1152	7		ATG	TAA
*trnS2(UCN)*	J	11,696–11,760	65	19	UGA		
*nad1*	N	11,780–12,718	939	0		ATT	TAA
*trnL1(CUN)*	N	12,719–12,787	69	**−**21	UAG		
*rrnL*	N	12,767–14,157	1391	0			
*trnV*	N	14,158–14,225	68	**−**1	UAC		
*rrnS*	N	14,225–15,022	798	0			
*A + T-rich*		15,023–15,502	480				

Strand of the genes is presented as J for majority and N for minority strand. IN, negative numbers indicate that adjacent genes overlap, positive numbers indicate intergenic sequences.

**Table 3.** Base composition and skewness of the *Laelia suffusa* mitogenome

	Size (bp)	A (bp)	G (bp)	T (bp)	C (bp)	A%	G%	T%	C%	A+T%	AT-Skew	GC-Skew
Whole genome	15,502	6,218	1,147	6,035	2,102	40.11	7.40	38.93	13.56	79.04	0.015	**−**0.294
PCGs	11,295	3,739	1,307	4,945	1,304	33.10	11.57	43.78	11.54	76.88	**−**0.139	0.001
tRNA genes	1,470	612	160	585	113	41.63	10.88	39.80	7.69	81.43	0.023	0.172
rRNA genes	2,189	912	254	920	103	41.66	11.60	42.03	4.71	83.69	**−**0.004	0.422
Control region	480	203	17	223	37	42.29	3.54	46.46	7.71	88.75	**−**0.047	**−**0.370

Gene rearrangement was detected in *L. suffusa* mitogenome compared with species from Hepialoidea and Nepticuloidea. In *L. suffusa* mitogenome, the control region gene order was *trnM, trnI*, and *trnQ*, which was different from the basal lepidopteran species (such as *Stigmella roborella*, *Ahamus yunnanensis,* and *Thitarodes gonggaensis*) (Fig. 2).

### Protein-Coding Genes and Codon Usage

The 13 PCGs were 11,295 bp in length, accounting for 72.86% of the complete *L. suffusa* mitogenome. Nine out of the 13 PCGs (*cox1*, *cox2*, *cox3*, *nad2*, *nad3*, *nad6, atp6*, *atp8,* and *cytb*) were scattered on the major strand, whereas four genes (*nad1, nad4*, *nad4l,* and *nad5*) were on the minor strand. The start and stop codons of all PCGs are shown in Table 2. The standard ATN start codon was used for most of the PCGs such as ATT for *nad1*, *nad2*, ATG for *cox3, nad4, nad4l, nad6, atp6, cytb,* and ATA for *cox2, nad3, nad5*, *atp8.* The only exception was in *cox1* which had CGA (arginine). Three out of the 13 PCGs used incomplete stop codons (T for *cox1, cox2,* and TA for *nad4*), and only *nad4* used incomplete termination codon (TA), whereas others used the typical stop codon (TAA).

**Fig. 3.**
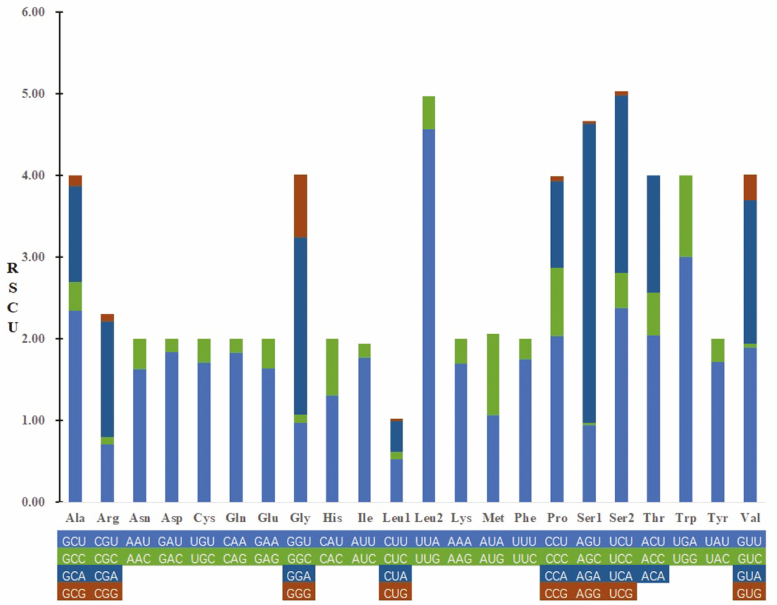
The relative synonymous codon usage in the mitogenome of *Laelia suffusa*. Codon families are provided on the *X*-axis.

**Fig. 4.**
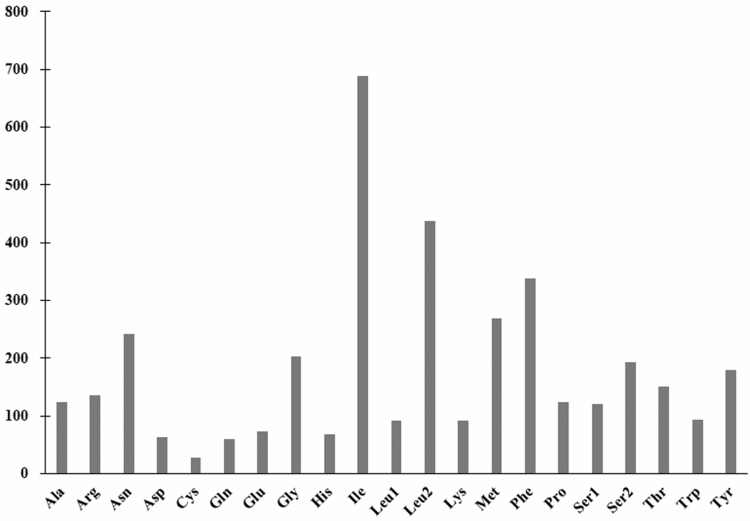
Codon distribution in *Laelia suffusa* mitogenome. Numbers to the left refer to the total number of the codon. Codon families are provided on the *X*-axis.

The average A + T content of the 13 PCGs was 76.88%, and the AT-skew was negative (−0.139), indicating more Ts than As (Table 3). The relative synonymous codon usage of *L. suffusa* was tested based on the 3,605 codons in 13 PCGs, and only codons ACG, UAA, and UAG were not presented (Table 4). The codon usage analysis demonstrated that isoleucine (I; Ile; 11.26%), leucine 2 (L; Leu2; 11.15%), phenylalanine (F; Phe; 8.21%), methionine (M; Met; 6.77%), and asparagine (N; Asn; 5.46%) were the most frequently used, whereas cysteine (C; Cys; 0.78%) was the least used (Figs. 3 and 4, Table 4). Codon distribution was consistent with other lepidopteran insects mitogenome.

**Table 4.** Codon usage of the protein-coding genes in *Laelia suffusa*

Codon (aa)	*n*	%	RSCU	Codon (aa)	*n*	%	RSCU
**UUU (F)**	**296**	**8.21**	**1.75**	UAU (Y)	155	4.30	1.72
UUC (F)	42	1.17	0.25	UAC (Y)	25	0.69	0.28
**UUA (L)**	**402**	**11.15**	**4.56**	UAA (*)	0	0.00	0.00
UUG (L)	36	1.00	0.41	UAG (*)	0	0.00	0.00
CUU (L)	47	1.30	0.53	CAU (H)	45	1.25	1.30
CUC (L)	8	0.22	0.09	CAC (H)	24	0.67	0.70
CUA (L)	33	0.92	0.37	CAA (Q)	54	1.50	1.83
CUG (L)	3	0.08	0.03	CAG (Q)	5	0.14	0.17
**AUU (I)**	**406**	**11.26**	**1.77**	**AAU (N)**	**197**	**5.46**	**1.63**
AUC (I)	39	1.08	0.17	AAC (N)	45	1.25	0.37
**AUA (M)**	**244**	**6.77**	**1.06**	AAA (K)	78	2.16	1.70
AUG (M)	25	0.69	1.00	AAG (K)	14	0.39	0.30
GUU (V)	74	2.05	1.89	GAU (D)	58	1.61	1.84
GUC (V)	2	0.06	0.05	GAC (D)	5	0.14	0.16
GUA (V)	69	1.91	1.76	GAA (E)	60	1.66	1.64
GUG (V)	12	0.33	0.31	GAG (E)	13	0.36	0.36
UCU (S)	91	2.52	2.37	UGU (C)	24	0.67	1.71
UCC (S)	17	0.47	0.44	UGC (C)	4	0.11	0.29
UCA (S)	83	2.30	2.17	UGA (W)	85	2.36	3.00
UCG (S)	2	0.06	0.05	UGG (W)	9	0.25	1.00
CCU (P)	63	1.75	2.03	CGU (R)	16	0.44	0.71
CCC (P)	26	0.72	0.84	CGC (R)	2	0.06	0.09
CCA (P)	33	0.92	1.06	CGA (R)	32	0.89	1.41
CCG (P)	2	0.06	0.06	CGG (R)	2	0.06	0.09
ACU (T)	77	2.14	2.04	AGU (S)	36	1.00	0.94
ACC (T)	20	0.55	0.53	AGC (S)	1	0.03	0.03
ACA (T)	54	1.50	1.43	AGA (S)	83	2.30	3.66
ACG (T)	0	0.00	0.00	AGG (S)	1	0.03	0.04
GCU (A)	72	2.00	2.34	GGU (G)	49	1.36	0.97
GCC (A)	11	0.31	0.36	GGC (G)	5	0.14	0.10
GCA (A)	36	1.00	1.17	GGA (G)	110	3.05	2.17
GCG (A)	4	0.11	0.13	GGG (G)	39	1.08	0.77

In total, 3,605 codons were analyzed. RSCU stands for relative synonymous codon usage. * stands for termination codon. The codons in bold were the most commonly used in the mitogenome of *L. suffusa*.

### Transfer RNAs and ribosomal RNAs

The *L. suffusa* mitogenome contained 22 *tRNA* genes that were scattered throughout the entire genome. The *tRNA* length varied from 64 bp (*trnT*) to 71 bp (*trnK*), and this was consistent with previously reported Lepidoptera mitogenomes. In total, 14 *tRNA*s were encoded on the major chain and the rests were on the minor chain. The combined sequence of the 22 *tRNA*s was 1,470 bp, and the A + T content was 81.43% with a positive AT-skew and negative GC-skew. The A + T content of the two *rRNAs* genes was slightly higher than that in *tRNA*s, accounting for 83.69% of the total 2,189-bp sequence (Table 3).

**Fig. 5.**
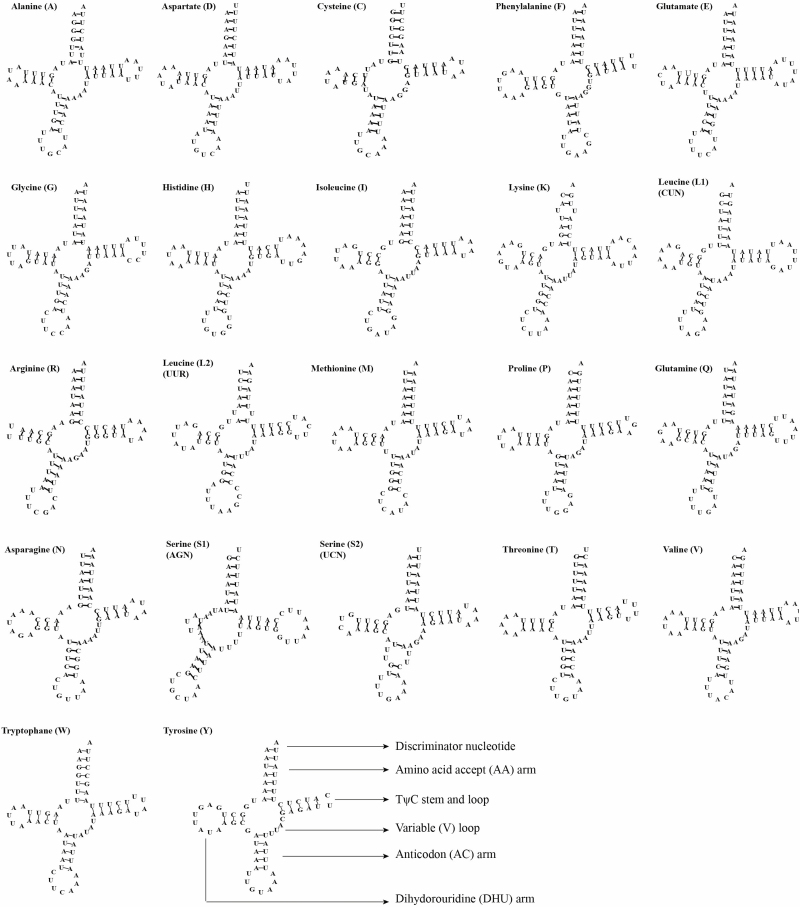
Putative secondary structures for the tRNA genes of the *Laelia suffusa* mitogenome.

Almost all of the 22 tRNAs formed the typical clover-leaf secondary structures, except for the *trnS1(AGN)* (Fig. 5). The dihydorouridine (DHU) arm of *trnS1* formed a loop instead of a couple of paired bases. The amino acid acceptor (AA) arm length of the *tRNA*s was unified (7 bp). Several anticodons (AC) loops comprised of six nucleotides except for five *tRNA*s (*trnC, trnI, trnK, trnR,* and *trnN*) whose AA stem was 7 bp, as well as *trnS1(AGN)* with eight nucleotides. The TψC (T) length and its arm ranged from 2 to 9 bp and 4 to 5 bp, respectively. The DHU stem length varied from 3 to 4 bp except in *trnS1(AGN)* with 2 to 8 bp. The AC arms were all 5 bp except for *trnL2(UUR)* whose AC stem was 4 bp.

Both *rRNA* genes were encoded on the N strand. The large ribosomal RNA (*rrnL*), with a length of 1,391 bp was located between *trnL1* and *trnV*, whereas the small ribosomal RNA (*rrnS*), with a length of 798 bp, was located between *trnV* and the control region (Table 2). The *rrnL* and *rrnS* showed significant high A+T content (83.03 and 84.84%, respectively) bias; however, the AT-skew was different (−0.020 and 0.022).

### Overlapping and Noncoding Regions

In total, 10 overlapping regions were detected in the *L. suffusa* mitogenome which varied between 1 and 41 bp. The longest overlapping region was 41 bp, located between the *trnY* and *cox1* (Table 2). There were 19 intergenic spacers scattered throughout the *L. suffusa* mitogenome and ranging from 2 to 73 bp. The longest spacer region was detected to be located between gene *trnQ* and *nad2*, which was an A + T-rich region.

**Fig. 6.**
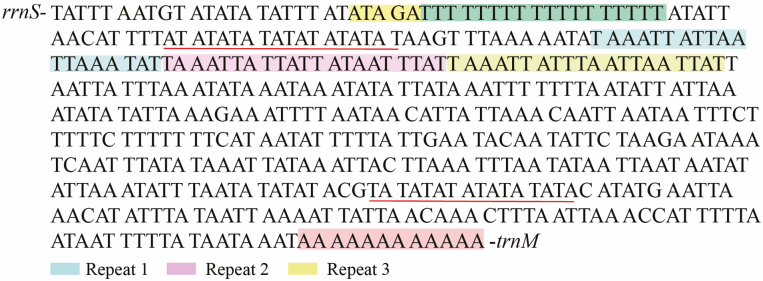
Features present in the AT-rich region of the *Laelia suffusa* mitogenome. Colored nucleotides indicate the ATATA motif (yellow), the poly-T stretch (green) and microsatellite A/T repeat sequences were underlined. Three tandem repeats are indicated in different colors.

The control region (A + T-rich region) of *L. suffusa* located between the *rrnS* and *trnM* genes was the longest noncoding region (480 bp) in the entire mitogenome (Fig. 1, Table 2). This highest AT content (88.75%) was also found in this region, with a negative AT-skew (−0.047) and GC-skew (−0.370). In the *L. suffusa* mitogenome, an ATAGA motif close to gene *rrnS* was also detected and found to be a conserved feature of lepidopteran’s mitogenomes (Cameron and Whiting 2008). An 18-bp poly-T stretch following the ATAGA motif and a 12-bp poly-A string followed by *trnM* were reported. Three microsatellite-like repetitive elements were also found (Fig. 6).

### Phylogenetic Analyses

**Fig. 7.**
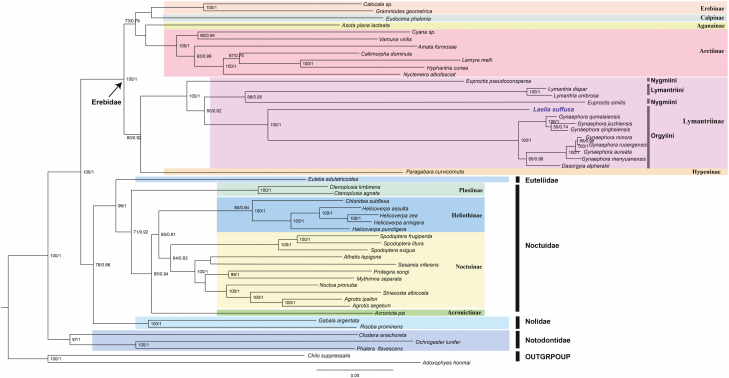
Phylogenetic tree inferred from nucleotide sequences of 13 PCGs of using the ML and BI analysis. Numbers on the branches are ML bootstrap support and BI posterior probability.

**Fig. 8.**
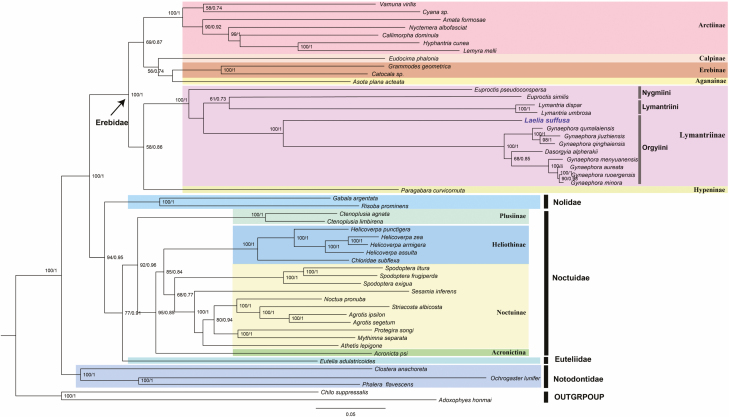
Phylogenetic tree inferred from nucleotide sequences of 37 genes (13 PCGs + 22tRNA + 2 rRNA) using the ML and BI analysis. Numbers on the branches are ML bootstrap support and BI posterior probability.

The phylogenetic relationships among the 52 Lepidoptera species (Table 1) were reconstructed based on the two nucleotide sequences (13 PCGs and 37 genes) datasets using the Maximum Likelihood (ML) and Bayesian Inference (BI) approaches. Besides, *Chilo suppressalis* and *Adoxophyes honmai*, which belong to Pyraloidea and Tortricoidea, respectively, were used as outgroups. All the other 50 species were from Noctuoidea. Phylogenetic analysis based on different algorithms (ML and BI analysis) showed approximately identical topologies. Phylogenetic relationships based on the two nucleotide datasets are shown in Figs. 7 and 8. Noctuoidea species were clustered into five families (Erebidae, Noctuidae, Euteliidae, Nolidae, and Notodontidae). The monophyly of Erebidae was well supported based on the topology of the phylogenetic tree. Within the Erebidae, the subfamilies Lymantriinae, Arctinae, and Erebinae were also monophyletic (Figs. 7 and 8). The phylogenetic relationships obtained were based on the two datasets (13 PCGs and 37 genes) and they were found to be consistent within the Noctuidae. All of the four families (Plusiinae, Heliothinae, Noctuinae, and Acronictinae) in Noctuidae were all monophyletic clades.

Besides the phylogenetic relationships among the Noctuoidae families were reconstructed, the results revealed three main clades in Lymantriinae: Nygmiini + (Lymantriini + Orgyiini). *Laelia suffusa*, *Dasorgyia alpherakii*, and seven species of *Gynaephora* clustered in one lineage, which supported that *L. suffusa* belongs to Orgyiini. Moreover, there was no clear threshold between *Gynaephora* and *Dasorgyia* due to the mixture of the two genera. Within the Lymantriinae, Orgyiini was the most closely related to Lymantriini, and Nygmiini was a sister group. Further, among these subfamilies, Hypeninae was closely related to Lymantriinae even though the support was not very strong.

## Discussion

Among the 13 species of the Lymantriinae subfamily, the *L. suffusa* mitogenome length (15,502 bp) was slightly smaller than the others, except for *E. pseudoconspersa* (15,461 bp) and *E. similis* (15,437 bp). The difference was attributed to the variable sequences in the noncoding regions and the control region (CR) (Rand 1993, McKnight and Shafer 1997). Moreover, the positive AT-skew (0.015) was associated with a higher frequency of guanine compared with thymine, which is a common phenomenon in Noctuoidea mitogenomes.

Gene rearrangement was also detected in *L. suffusa* when compared with the basal taxon of Lepidoptera, such as Hepialoidea and Nepticuloidea (Fig. 2). In lepidopteran mitogenomes, gene rearrangement mainly occurred in three *tRNA* genes: *trnM, trnI, trnQ* (Timmermans and Vogler 2012), as identified in the *L. suffusa* mitogenome. Only two mitogenomes from Nepticuloidea were chosen for the gene rearrangement analysis, and the results were inconsistent. *Astrotischeria* sp. showed a similar pattern of *L. suffusa.* However, *Thitarodes gonggaensis* (The other species chosen from Nepticuloidea) shared similar gene order with Hepialoidea, which was considered to be the ancestral pattern (CR*-trnI-trnQ-trnM*) (Fig. 2; Zhu et al. 2017, Yang et al. 2019). Based on the phylogenetic results of Mitter et al. (2017), this gene rearrangement likely occurred after the diverging of Hepialoidea superfamily from other lepidopteran lineages. Many models and hypotheses have been used to explain the mitogenome gene rearrangement, such as the tandem duplication-random loss (TDRL) model (Boore 2000), the duplication-nonrandom loss model (Lavrov et al. 2002), and the recombination (Cantatore et al. 1987) and illicit priming of replication by tRNA genes (Dowton et al. 2009). The gene rearrangement of *L. suffusa* mitogenome can be well explained by a combination of the TDRL model and recombination.

The AT-skew value of the 13 PCGs was −0.139 in the *L. suffusa* mitogenome was lower than that reported in previously sequenced Lymantriinae mitogenomes. Meanwhile, the slightly positive GC-skew of 0.001 was also higher than that reported in other species. The gene *cox1* had a different start codon (CGA) compared with the other 12 protein-coding genes and this is similar to other Lymantriinae insects (Yuan et al. 2018). The incomplete stop codons were observed in Lepidoptera mitogenomes (Ronquist and Huelsenbeck 2003, Stamatakis 2006, Ronquist et al. 2012, Yuan et al. 2018). In the newly sequenced mitogenome, three genes were associated with the incomplete stop codons. For example, *cox1* and *cox2* used single T and *nad4* used TA. The common interpretation for the high frequency of the TAA stop codon is that the TAA terminator is created via post-transcriptional polyadenylation (Ojala et al. 1981). All *tRNA* genes were predicted to have formed the typical clover-leaf structure, except for *trnS1*, whose dihydorouridine (DHU) arm formed a loop instead of a couple of paired bases. This is a universal phenomenon occurring in insect mitogenomes and metazoan mitogenomes (Wolstenholme 1992, Ronquist and Huelsenbeck 2003, Stamatakis 2006, Ronquist et al. 2012, Tang et al. 2017). Furthermore, the intergenic spacer located between *atp6* and *atp8* contained a conserved seven-nucleotide structure (ATGATAA), which is also reported in other lepidopteran mitogenomes. This implies a stable evolutionary structure and potential molecular marker for Lepidoptera of mitogenome (Stamatakis 2006, Ronquist et al. 2012). Noctuoidea is the largest in the Lepidoptera superfamily and includes 4,200 genera and up to 42,400 species (Kitching and Rawlins 1998). Currently, it was widely believed that there were six families in Noctuoidea: Oenosandridae (include 4 genera), Notodontidae (704 genera), Erebidae (1,760 genera), Euteliidae (29 genera), Nolidae (186 genera), and Noctuidae (704 genera) (Kitching and Rawlins 1998; Zahiri et al. 2011, 2012). However, a change in the molecular markers or sampling taxon might lead to different phylogenetic structures. The phylogenetic relationship among the families within Noctuoidea has been debated for a long time (Liu et al. 2016). The monophyly of Noctuidae was confirmed based on the morphological characteristics of the adult and larva; however, the relationships among subfamilies and genera showed poor resolutions (Speidel et al. 1996, Fibiger et al. 2005, Zahiri et al. 2011). Zahiri et al. (2011) revealed that the phylogenetic relationship of Noctuidae was as follows: (Notodontidae + (Euteliidae + (Noctuidae + (Erebidae + Nolidae). This study was based on a single mitochondrial gene (*cox1*) and seven nuclear genes. However, different taxon and molecular markers have been identified various phylogenetic relationships, including (Notodontidae + (Noctuidae + (Nolidae + (Erebidae +Euteliidae)))) (Mitchell et al. 2006); (Notodontidae + (Nolidae + (Noctuidae + (Erebidae +Euteliidae)))) (Fibiger et al. 2005); (Notodontidae + (Erebidae + (Noctuidae + (Euteliidae + Nolidae)))) (Regier et al. 2017).

In this study, the robust phylogenetic relationships obtained based on the two datasets were similar. The relationships of Noctuoidea were described as (Notodontidae + (Erebidae + (Nolidae + (Noctuidae +Euteliidae). These results were consistent with those reported recently, supporting that Noctuoidae and Euteliidae were the closest taxa, and the Notodontidae was the ancestral family in Noctuoidea (Zahiri et al. 2011, Yang et al. 2019).

The concatenated nucleotide sequences of the 13 PCGs and 37 genes using ML and BI methods provided a well-supported outline of Erebidae. The 25 species of Erebidae were divided into two groups: Erebinae + Calpinae + Aganainae +Arctiinae, which were clustered as one group, and the other two subfamilies Lymantriinae and Hypeninae clustered in the other group and these findings were similar to those reported in previous studies (Zahiri et al. 2011, 2012; Wang et al. 2015). Initially, *L. suffusa* was classified into the family Lymantriidae, which later became Erebidae, and subfamily Lymantriinae (Mitter et al. 2017, Regier et al. 2017). These findings provide strong evidence for the classification of *L. suffusa,* which belongs to the Orgyiini of Lymantriinae. All the 13 species of Lymantriinae clustered into a stable monophyletic group which was supported with strong evidence (PP = 1.0, BS = 100). There were three sister clades included in this group: the first one included *L. suffusa* as a sister group of the *Dasorgyia alpherakii* and the genus *Gynaephora,* which was well supported (PP = 1.0, BS = 100) by ML and BI analysis; and in the second clade, *Euproctis similis* was at the basal position and a sister to *Lymantria dispar* + *Lymantria umbrosa;* in the last clade, a single species *Euproctis pseudoconspersa,* was the basal taxa of Lymantriinae. However, the evolutionary history and phylogenetic relationship of Erebidae and Lymantriinae have attracted great attention and remained unclear (Kitching and Rawlins 1998, Mitter et al. 2017, Regier et al. 2017). Therefore, more advanced studies using larger sample sizes and genetic information are imperative to solve the phylogenetic relationship of Noctuoidea.
